# Physiologic biventricular repair in a patient with unrepaired adult congenital heart disease with severe cyanosis

**DOI:** 10.1016/j.xjtc.2022.07.020

**Published:** 2022-08-08

**Authors:** Hiroyuki Suzuki, Yosuke Kuroko, Yasuhiro Kotani, Shingo Kasahara

**Affiliations:** Department of Cardiovascular Surgery, Okayama University, Okayama, Japan

**Figure:**
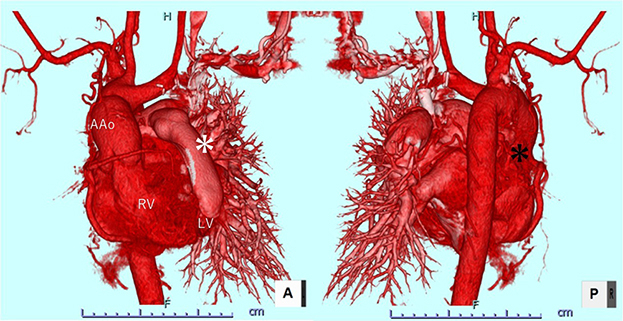
Postoperative contrast computed tomography image. *Black asterisk* indicates occluded right PA; *white asterisk* indicates LV-PA conduit.

**Central Message**

Physiologic biventricular repair may improve quality of life in patients with cyanotic adult congenital heart disease and reasonable anatomic conditions.

The optimal treatment for adult patients with unrepaired congenital heart disease is under debate. We report a case of unrepaired double outlet right ventricle (DORV) with severe cyanosis in a patient who underwent biventricular conversion (BVC) in adulthood.

## Clinical Summary

A 37-year-old man, who was born with DORV with remote ventricular septal defect (VSD), pulmonary atresia, and persistent left superior vena cava (PLSVC), underwent left original Blalock-Taussig shunt (BTS) when he was 8 months old, followed by additional right modified BTS at age 6 years, and central shunt, right pulmonary artery (PA) plasty, and right superior vena cava division for right PA stenosis at age 8 years. Although the left BTS was patent, the other shunts were later occluded. Moreover, the right PA was occluded such that the right lung was supplied only by the aortopulmonary collateral arteries. For these reasons, he was not a Fontan candidate.

**Figure 1:**
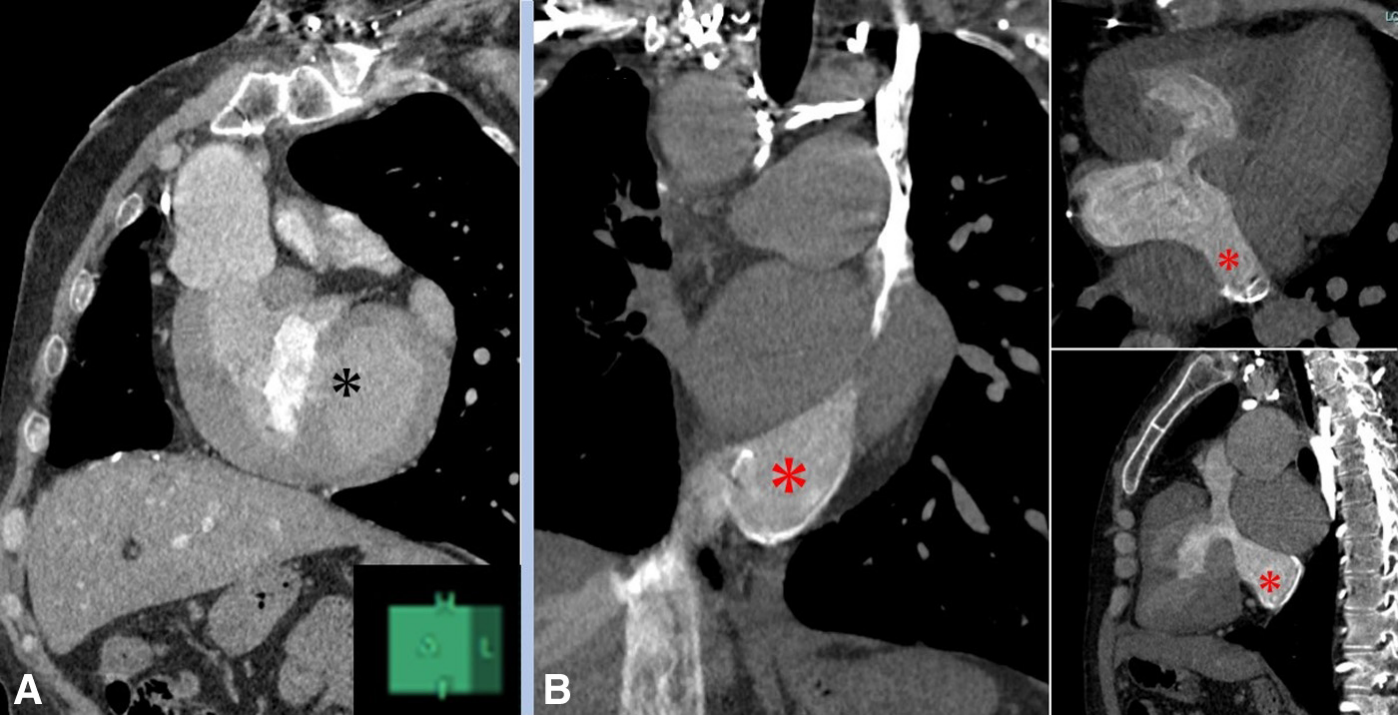
Preoperative contrast computed tomography images. A, Remote ventricular septal defect (*black asterisk*). B, Dilated coronary sinus (*red asterisk*) in coronal, horizontal, and sagittal planes.

**Figure 2:**
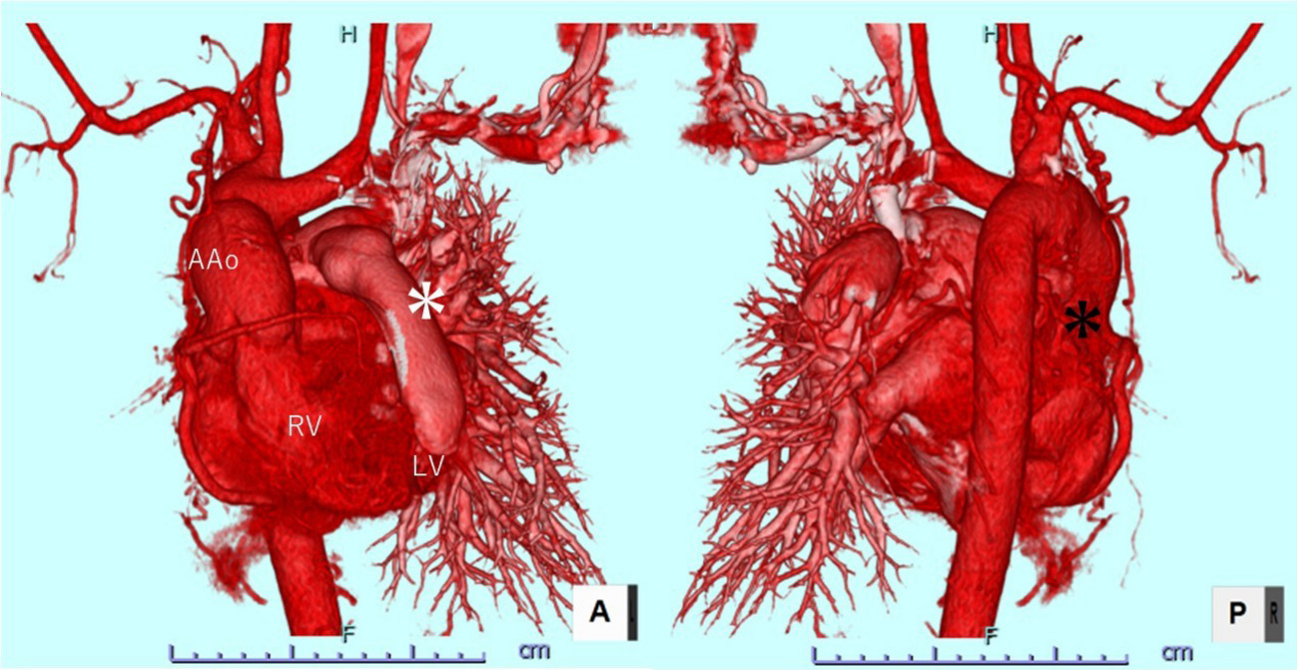
Postoperative contrast computed tomography image. *Black asterisk* indicates occluded right pulmonary artery; *white asterisk* indicates left ventricle-pulmonary artery conduit. *AAo*, Ascending aorta; *RV*, right ventricle; *LV*, left ventricle.

**Figure E1:**
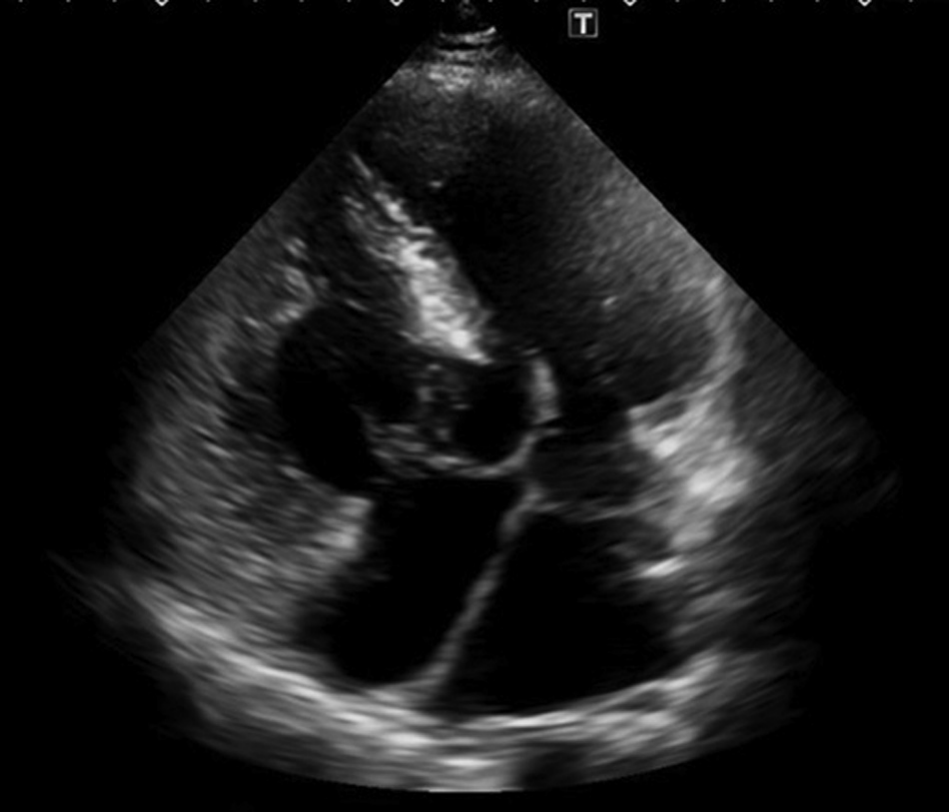
This is a 4-chamber view of preoperative echocardiography. The tricuspid and mitral valve sizes are 26 mm (*z* score, –1.02) and 33 mm (*z* score, 0.90), respectively.

In adulthood, with dyspnea on exertion due to severe hypoxemia with arterial oxygen saturation of 81% on room air, he was forced to stay at home (New York Heart Association functional class III). Echocardiography revealed no valvular stenosis or regurgitation more than moderate, and tricuspid and mitral valve sizes were 26 mm (*z* score, –1.02) and 33 mm (*z* score, 0.90), respectively, which were adequate for BVC (Figure E1). On cardiac catheterization, right ventricle (RV) pressure was 72/4 (27 mm Hg), and volumetry with cardiac magnetic resonance imaging resulted in RV and LV end-diastolic volume index of 50.3 mL/m^2^ (*z* score, –2.5) and 76.2 mL/m^2^ (*z* score, –0.2), and ejection fractions of 50% and 67%, respectively. On computed tomography, the VSD was located too far from the aortic valve to reroute (Figure 1, *A*). We planned BVC to improve hypoxia, which leads to exercise intolerance. He underwent atrial switch, VSD closure, LV-PA reconstruction with an expanded polytetrafluoroethylene conduit with bulging sinuses and a fan-shaped valve, and BTS division (Video 1). The LV incision site was located lower to avoid the conduit being compressed by the sternum and heart. Because the systemic venous return originated from the inferior vena cava and the PLSVC through the dilated coronary sinus (CS) (Figure 1, *B*), atrial switch was completed by unroofing the CS and reconstructing the inferior limb with an artificial graft. Postoperatively, he had no dyspnea on exertion (New York Heart Association functional class II) with arterial oxygen saturation of 97% on room air. Apart from pacemaker implantation due to complete atrioventricular block, the patient's postoperative course was uneventful, including his computed tomography image (Figure 2), and he was discharged. Postoperative cardiac catheterization showed aorta 106/77 (87) mm Hg, PA 40/17 (24) mm Hg, LV 38/1 (10) mm Hg, and IVC 9 mm Hg. Cardiopulmonary exercise testing after 12 weeks of surgery showed elevated peak volume of oxygen consumption (Vo_2_) compared to preoperative levels, from 12.7 to 16.5 mL/kg/min. Written consent was obtained from the patient to publish this case report and associated figures. The institutional review board of Okayama University approved this case report (Approval No. CRB6180001; March 23, 2020).

## Discussion

The quality of life of patients with unrepaired cyanotic congenital heart disease is believed to be limited by hypoxemia, and low peak Vo_2_ indicates a correlation with symptoms and exercise tolerance.^1^ Najm and colleagues^2^ reported the new biventricular repair concept of ventricular switch, using the LV as the subpulmonary ventricle to resolve hypoxemia in such patients. We also believe that the correction of severe hypoxia by BVC improved exercise tolerance, as evidenced by the elimination of symptoms and elevated peak Vo_2_ in cardiopulmonary exercise testing. Winter and colleagues^3^ reported that exercise training improves exercise capacity in adult patients with a morphological RV that sustains the systemic circulation. This indicates, that in this patient in the same status, the elimination of symptoms due to BVC would promote exercise in daily life, which in turn improves exercise tolerance.

When performing atrial switch in patients with PLSVC or CS enlargement, it is effective to unroof the CS. However, we have to mention the atrioventricular block, which was believed to be developed by an incision of CS unroofing. The prominently enlarged CS may have made the atrioventricular node and its feeding artery deviate from their original positions. Therefore, in anticipation of the above, the incision has to be made in a way that maintains sufficient distance from the atrioventricular node. Although we cannot completely rule out the possibility of heart block by VSD closure, we believe that the CS incision is more likely to be the cause.

When a morphological RV is used to propel blood through the systemic circulation, instead of a left ventricle, it may lead to heart failure in the long-term.^4^ Although this case had favorable short-term results, the patient needs careful follow-up. This case highlights the importance of considering BVC in patients with cyanotic adult congenital heart disease and reasonable anatomic conditions.

## Supplementary Data

Video 1 This video shows BVC, which includes atrial switch, VSD closure, left ventricular-pulmonary artery reconstruction with an expanded polytetrafluoroethylene conduit with bulging sinuses and a fan-shaped valve, and BTS division. Video available at: https://www.jtcvs.org/article/S2666-2507(22)00433-3/fulltext.

## References

[1] Gläser S., Opitz C.F., Bauer U., Wensel R., Ewert R., Lange P.E. (2004). Assessment of symptoms and exercise capacity in cyanotic patients with congenital heart disease. Chest.

[2] Najm H.K., Karamlou T., Ahmad M., Hassan S., Yaman M., Stewart R. (2020). Biventricular conversion in unseptatable hearts: “ventricular switch.”. Semin Thorac Cardiovasc Surg.

[3] Winter M.M., van der Bom T., de Vries L.C.S., Balducci A., Bouma B.J., Pieper P.G. (2012). Exercise training improves exercise capacity in adult patients with a systemic right ventricle: a randomized clinical trial. Eur Heart J.

[4] Piran S., Veldtman G., Siu S., Webb G.D., Liu P.P. (2002). Heart failure and ventricular dysfunction in patients with single or systemic right ventricles. Circulation.

