# Supplementary figures and images for: Physiologic biventricular repair in a patient with unrepaired adult congenital heart disease with severe cyanosis
Source: JTCVS Tech. 2022 Aug 8;15:220–3. doi: 10.1016/j.xjtc.2022.07.020 (PMC9579854; doi:10.1016/j.xjtc.2022.07.020)

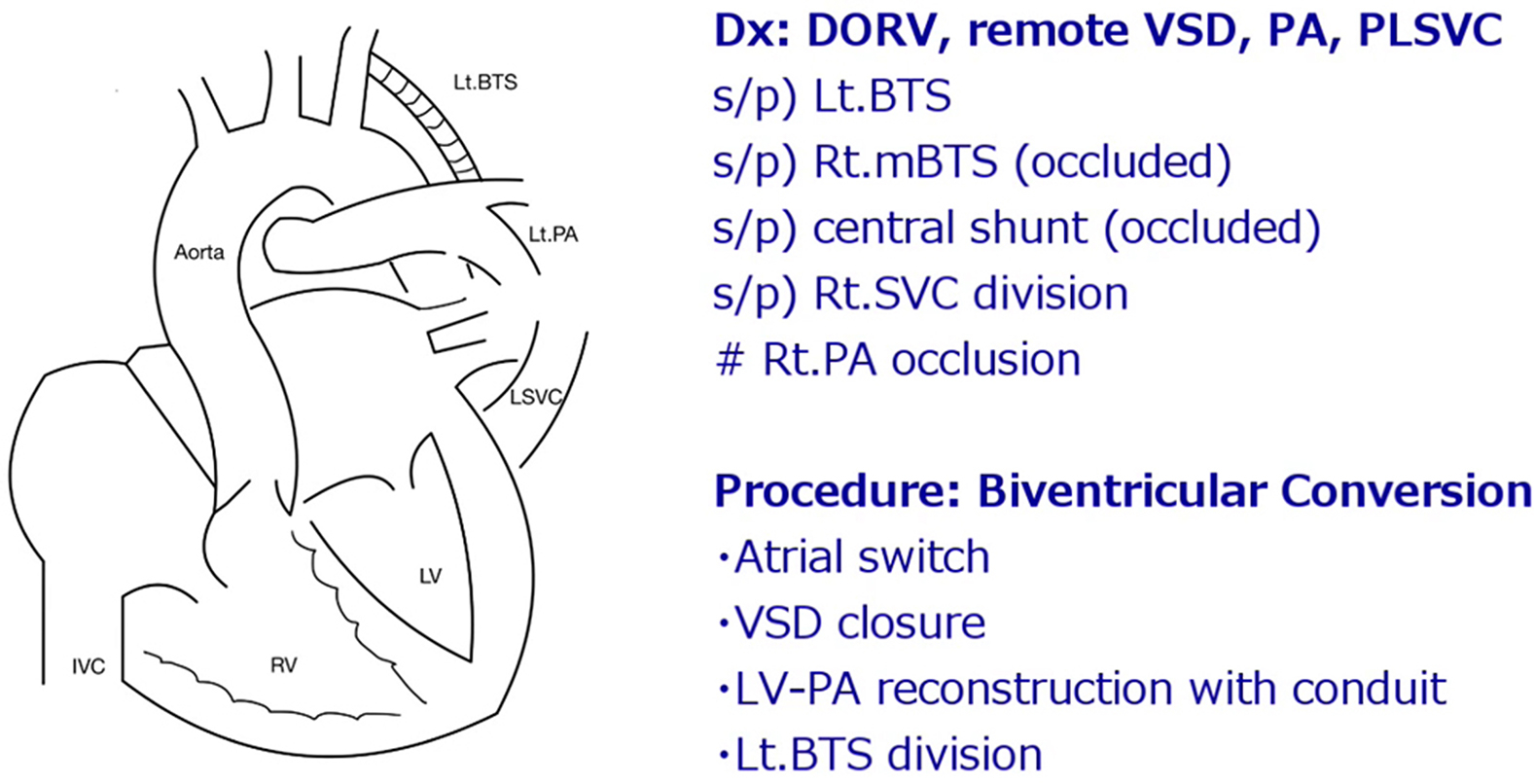

Supplement: Video 1 — This video shows BVC, which includes atrial switch, VSD closure, left ventricular-pulmonary artery reconstruction with an expanded polytetrafluoroethylene conduit with bulging sinuses and a fan-shaped valve, and BTS division. Video available at: https://www.jtcvs.org/article/S2666-2507(22)00433-3/fulltext. [file fx2.jpg]
